# Pretibial myxedema in a euthyroid patient: a case report

**DOI:** 10.1186/s13044-021-00096-z

**Published:** 2021-03-01

**Authors:** Rediet Ambachew, Tizita Yosef, Aklilu M. Gebremariam, Lishan Demere, Theodros Aberra, Getahun Tarekegn, Ahmed Reja

**Affiliations:** 1 Department of Endocrinology, Addis Ababa University, College of health sciences, Addis Ababa, Ethiopia; 2 Department of Dermatology and Venereology, Addis Ababa University, College of health sciences, Addis Ababa, Ethiopia; 3 Department of Pathology, Armauer Hansen Research Institute, Addis Ababa, Ethiopia

**Keywords:** Pretibial myxedema, Euthyroid

## Abstract

**Background:**

Pretibial myxedema also known as localized myxedema, thyroid dermopathy, or infiltrative dermopathy and rarely as localized mucinosis is an infrequent manifestation of Graves’ disease. It can appear before, during, or after the thyrotoxic state. Euthyroid pretibial myxedema is a rare presentation with few case reports in the literature. This case highlights the importance of considering pretibial myxedema when characteristic skin lesions are observed in a euthyroid patient.

**Case presentation:**

A 72-year old male Ethiopian patient with a very rare presentation of biopsy-proven pretibial myxedema in a euthyroid state without history of thyroid disease and absence of thyroid autoimmune markers. Resolution of skin lesion was achieved after topical corticosteroid application.

**Conclusion:**

Absence of history of thyroid disorder and normal thyroid function tests should not exclude the diagnosis of pretibial myxedema.

## Background

Pretibial myxedema forms the third component of the classical triad of Graves’ disease (goiter, ophthalmopathy, pretibial myxedema) [1]. It is not restricted to the pretibial area but may extend to the ankle and dorsum of the foot and may present on the elbows, knees, upper back and neck [1].

Pretibial myxedema used to be seen in up to 5% of patients with Graves’ disease and 15% of patients who have both Graves’ disease and ophthalmopathy [2] but the incidence of pretibial myxedema has declined substantially, probably because the diagnosis of Graves’ hyperthyroidism is now established much earlier and anti-thyroid therapy is initiated sooner [3].

Pretibial myxedema also occurs, very rarely, in patients with no past or present thyroid dysfunction and in patients with chronic autoimmune thyroiditis [4].

We report a rare case of a biopsy proven pretibial myxedema in a patient with normal thyroid function and absence of thyroid autoantibodies.

## Case report

A 72- year old man presented to the dermatology clinic with a six-month history of indurated skin lesion involving both shins. The initial lesion started as asymptomatic, erythematous papules, which slowly coalesced and formed an infiltrative indurated plaque. Over the ensuing months the lesion enlarged to cover the entire lower two-third of the pretibial region, causing associated edema, itching and discomfort. There was no history of pretibial trauma or insect bite. He had no self or family history of thyroid illness and no current symptoms of thyrotoxicosis or thyroiditis.

He was known to have type 2 diabetes mellitus for the last 20 years and hypertension for 10 years. His medications included insulin, enalapril, atorvastatin and aspirin.

On physical examination, his blood pressure was 140/90 mmHg and body mass index 31 kg/meter^2^.

There was no palpable thyroid swelling, signs of ophthalmopathy or acropachy. He had central obesity with waist circumference of 115 cm.

**Fig. 1:**
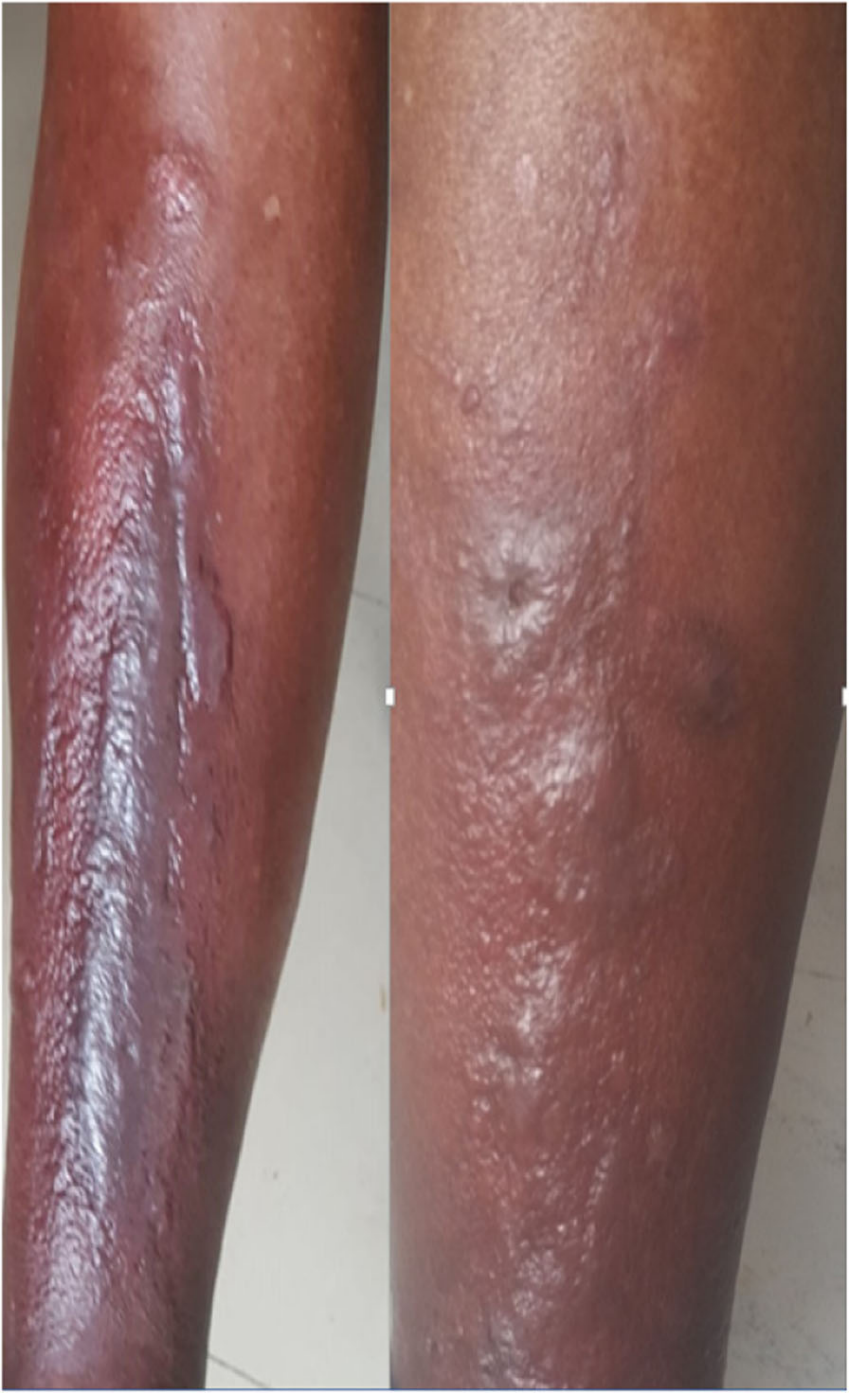
Indurated translucent papules and plaque, with erythematous and hyperpigmented background, non-pitting edema on the pretibial areas bilaterally with “peau d’orange” appearance

Dermatological examination revealed an indurated translucent papules and plaque with erythematous and hyperpigmented background, non-pitting edema on the pretibial areas bilaterally with “peau d’orange” appearance [Fig. 1]. He had acanthosis nigricans with skin tags over the posterior neck, arm pit and elbow.

In our patient pretibial myxedema, necrobiosis lipoidica diabeticorum, stasis dermatitis and scleroderma were considered as possible causes of his skin lesion.

His free T4 was 15.4 pmol/L [reference range: [11–21], total T4 303 nmol/L [41–350], total T3 0.05 nmol/L [0.01–0.06], free T3 2.6 pmol/L [2–4], TSH 2.4 uU/ml [0.27–4.2]. His thyroid function tests were repeated three times in 1 month interval and were within normal reference range. Complete blood count, liver function tests, electrolytes were within normal reference range. His fasting blood sugar was 170 mg/dl and HbA1c [glycated hemoglobin] was 10%. Renal function tests showed creatinine 1.7 mg/dl [0.5–1.04], urea 47 mg/dl [14–45] and estimated glomerular filtration rate [eGFR] 38 ml/min/1.73m^2^ and he had proteinuria on urinalysis.

His Anti TSH receptor antibody [TRAB] was 0.52 IU/L [0–1.75], anti-thyroid peroxidase (TPO-Ab) was 10.8 IU/ml [2–34] and anti-thyroglobulin was 126 IU/ml [< 115]. Basal serum cortisol with adrenocorticotropic hormone [ACTH] were within normal range.

Neck ultrasonography was unremarkable. Doppler ultrasound of the lower limbs revealed normal arterial and venous study. Echocardiography showed mild left ventricular hypertrophy with grade 1 diastolic dysfunction.

**Fig. 2:**
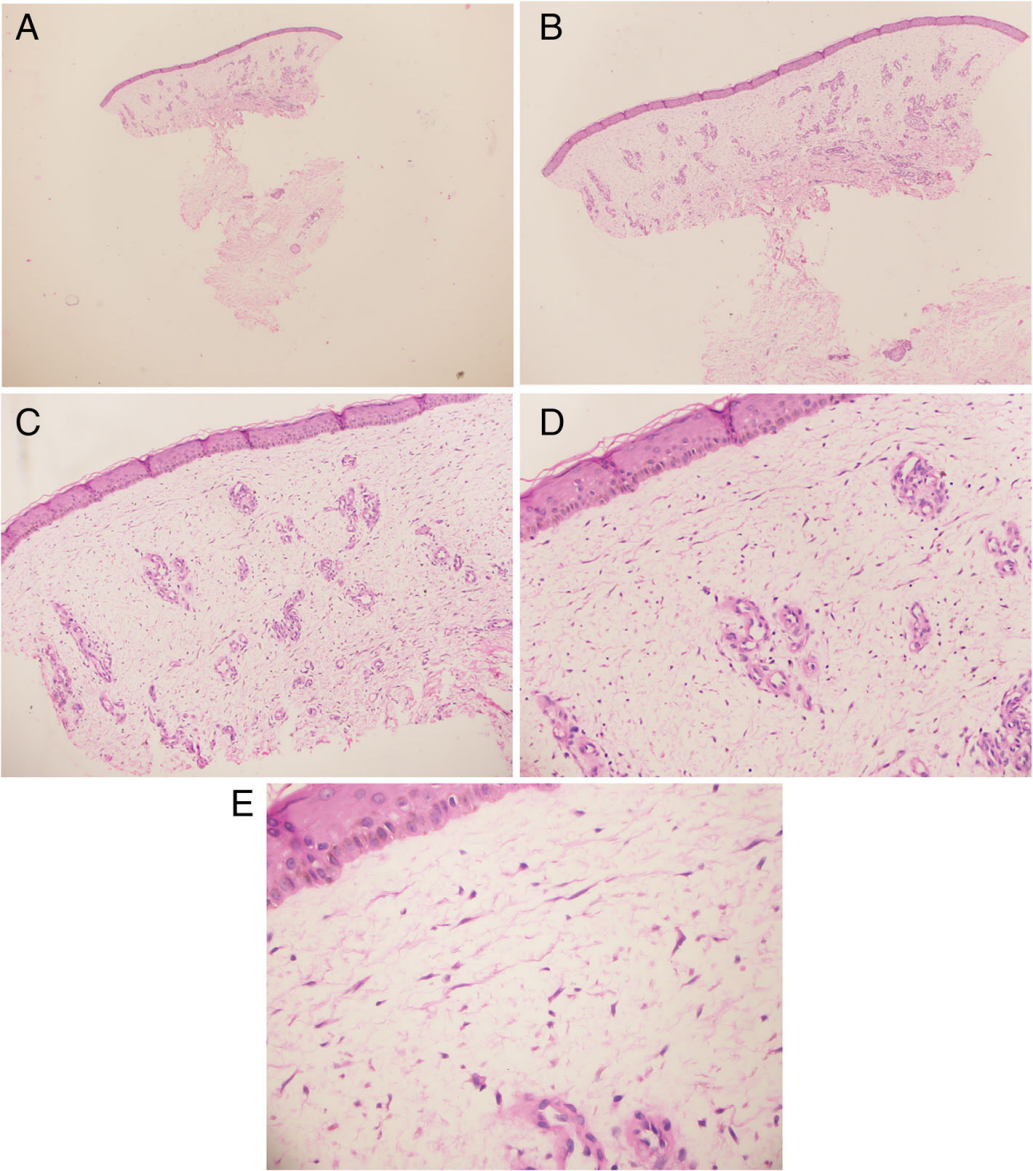
Histopathological features. There is pallor of the dermis 25x (**a**), 40x (**b**). Widely spaced thin collagen fibres in the dermis, a result of the increased amount of mucin in the interstitium 100x (**c**), 200x (**d**). Few stellate fibroblasts 400x (**e**). (Hematoxylin and eosin)

Skin biopsy showed histopathologic features consistent of myxedema demonstrated by abundant mucin deposition in both papillary dermis and reticular dermis between thin fibrils of collagen with no associated inflammatory changes [Fig. 2].

**Fig. 3:**
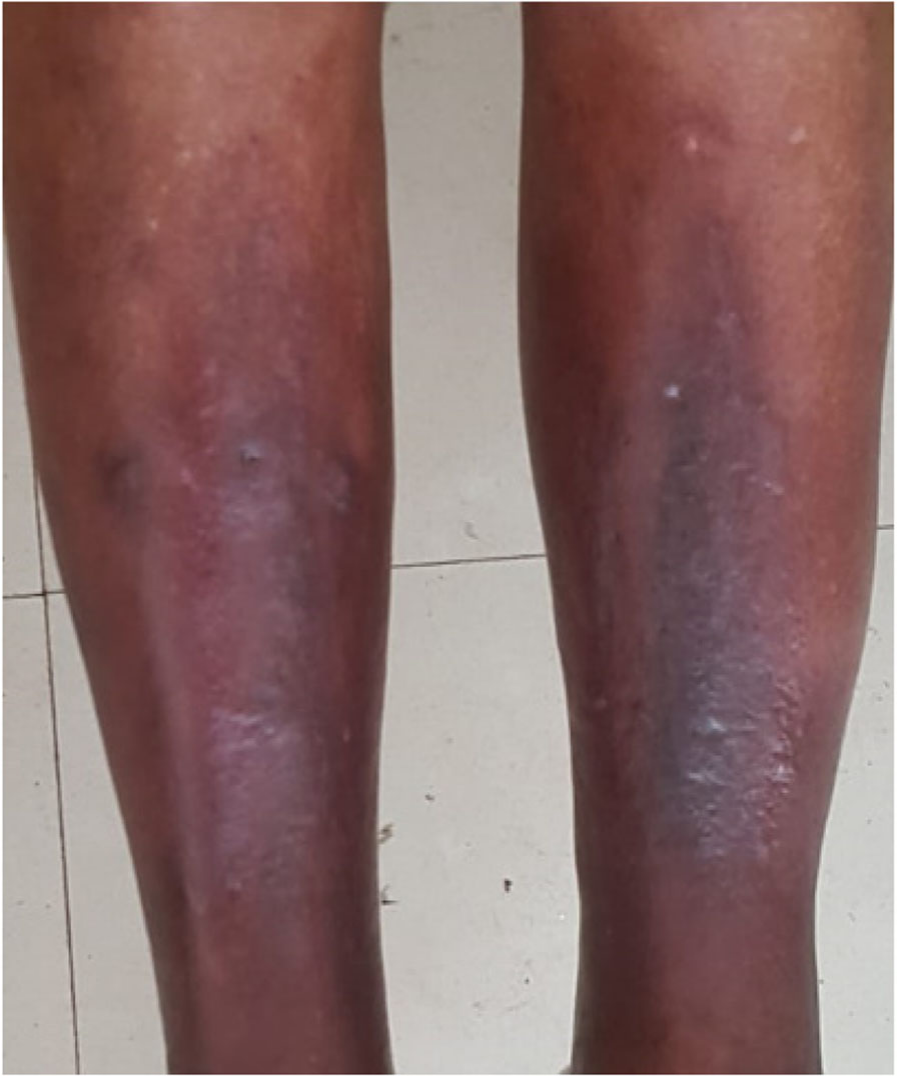
Improvement of the skin lesions - after treatment

The diagnosis of pretibial myxedema in a euthyroid state was confirmed and he was started on treatment with super potent topical corticosteroid under occlusion with significant clinical improvement [Fig. 3].

## Discussion

Pretibial myxedema arises as a result of the deposition of hyaluronic acid in the dermis and sub cutis under the stimulation of cytokines. The precise cause of this phenomenon remains uncertain. TRAB is present in the serum of most patients with pretibial myxedema (80–100%), but it has also been found in the serum of patients without pretibial myxedema [5].

Pretibial myxedema is usually asymptomatic, but pruritic or painful lesions have been reported with hyperhidrosis and hypertrichosis [4].

Most patients who develop pretibial myxedema have ophthalmopathy 6–12 months prior to dermopathy though rare cases of pretibial myxedema without ophthalmopathy have been reported [6].

Pretibial myxedema may resemble the skin lesions caused by necrobiosis lipoidica diabeticorum and inflammatory dermatoses such as stasis dermatitis, cutaneous mucinosis in patients with lupus erythematosus, dermatomyositis, and scleroderma, lichen amyloidosis and hypertrophic lichen planus [4].

The diagnosis of pretibial myxedema is based upon a history of thyroid disease and the characteristic clinical appearance of the skin lesion. Punch biopsy of the skin is done to make a definitive diagnosis in case of atypical presentation.

Among the variants, plaques and nodules are common with a favorable clinical response to topical and intralesional corticosteroid while elephantine and diffuse forms respond poorly to therapy [7].

Although myxedema does not seem to be correlated to the serum concentrations of T_3_ and T_4_, only few cases of euthyroid myxedema patients have been reported in the literature [8–12].

Similar to our case, ophthalmopathy, biochemical thyroid dysfunction and thyroid autoantibodies were absent in three reported cases of pretibial myxedema [9, 10, 12].

In conclusion, we describe a rare case of a biopsy-proven pretibial myxedema in a euthyroid patient. Though it is infrequent, pretibial myxedema should be considered when distinctive pretibial skin lesions are observed in a euthyroid patient even in the absence of thyroid autoimmune markers.

## Data Availability

Required patient data used in the case report are available from the corresponding Author on request.
